# Validating an Instrument for Measuring Newly Graduated Nurses’ Adaptation

**DOI:** 10.3390/ijerph20042860

**Published:** 2023-02-06

**Authors:** Hafidza Baharum, Aniza Ismail, Zainudin Awang, Lisa McKenna, Roszita Ibrahim, Zainah Mohamed, Nor Haty Hassan

**Affiliations:** 1 Department of Community Health, Faculty of Medicine, Universiti Kebangsaan Malaysia, Kuala Lumpur 56000, Malaysia; 2 Faculty of Business and Management, Universiti Sultan Zainal Abidin, Terengganu 21300, Malaysia; 3 School of Nursing and Midwifery, La Trobe University, Bundoora, VIC 3086, Australia; 4 Department of Nursing, Faculty of Medicine, Universiti Kebangsaan Malaysia, Kuala Lumpur 56000, Malaysia

**Keywords:** adaptation, organization, academic, personality, confirmatory factor analysis, validation studies

## Abstract

A long-established approach, Confirmatory Factor Analysis (CFA) is used to validate measurement models of latent constructs. Employing CFA can be useful for assessing the validity and reliability of such models. The study adapted previous instruments and modified them to suit the current setting. The new measurement model is termed NENA-q. Exploratory factor analysis (EFA) revealed the instruments of the NENA-q model formed a construct of the second order with four dimensions, namely organizational contribution (OC), academic institution contribution (AIC), personality traits (PT), and newly employed nurses’ adaptation (NENA). Researchers administered the questionnaires to a sample of 496 newly employed nurses working in hospitals under the Ministry of Health (MOH) for the confirmation of the extracted dimensions. The study performed a two-step CFA procedure to validate NENA-q since the model involves higher-order constructs. The first step was individual CFA, while the second step was pooled CFA. The validation procedure through confirmatory factor analysis (CFA) found the model achieved the threshold of construct validity through fitness index assessment. The model also achieved convergent validity when all average variance extracted (AVE) exceeded the threshold value of greater than 0.5. The assessment of the composite reliability (CR) value indicates all CR values exceeded the threshold value of 0.6, which indicates the construct achieved composite reliability. Overall, the NENA-q model consisting of the OC construct, AIC construct, PT construct, and NENA construct for CFA has met the fitness indexes and passed the measurements of the AVE, CR, and normality test. Once the measurement models have been validated through CFA procedure, the researcher can assemble these constructs into structural model and estimate the required parameter through structural equation modelling (SEM) procedure.

## 1. Introduction

What factors contribute to the successful transition of new nurses from students to registered nurses? Carleto et al. reported that new nurses who are able to adjust themselves, integrate, and socialize with a new organizational culture experience job satisfaction and work performance [1]. Job satisfaction and work competency were also reported as the driving factors for the new nurses to remain in their current profession [2]. Moreover, organizational socialization theory illustrates the importance of the adaptation stage, which new nurses need in order to ensure the success and smoothness of their transition [3]. This signifies that the transition from newly graduated nurses to professional nurses must be seen from the standpoint of adaptation for them to successfully pass the transition period.

In the nursing context, the adaptation process is the method by which new nurses employ conscious thought and have the option to incorporate themselves and their surrounding environments [4]. Some scholars have stated that adaptation is part of the professional socialization process [5]. This form of socialization refers to internal mechanisms whose key features are modified personal identities and different ways of understanding the self. These characteristics affect new nurses’ perceptions of and responses to different scenarios [6]. Adaptation is divided into three stages and classified into two dimensions: professional adaptation and social adaptation. The first and second stages refer to professional adaptation, while the third stage is concerned with social adaptation. In the first stage, the new recruit becomes acquainted with, introduces themselves to, and incorporates the organizational environment. The second stage promotes the use of knowledge, philosophy, and technical aspects needed to perform specific roles and responsibilities. Finally, the newcomer is integrated into a work group [7].

Newly employed nurses who have the ability to adapt effectively and achieve equilibrium between the various factors they face are proven to be independent and efficient in implementing nursing practice [8,9]. As a consequence, researchers believe that emphasizing effective adaptation among new nurses could perhaps assist them to avoid transition shock and succeed during the challenging transition period. Therefore, this study was conducted to identify the main factors that help newly graduated nurses in Malaysia make a quick adjustment and adapt to a challenging work environment. The researcher divided the driven adaptation factors into three main domains, namely, organizational contributions, academic institution contributions, and personality traits toward newly employed nurses’ adaptation.

Newly Employed Nurses Adaptation Questionnaires (NENA-q)

Based on the Roy Adaptation Model, nursing goals are accomplished by determining the behavioral responses and other variables that impact the study subjects’ adaptive abilities [4]. This principle is being conceptualized in the evaluation phase of this study to determine the contributing factors to new nurses’ adaptation to the work environment. In this assessment, a person’s behavior towards external stimuli is associated with four adaptive modes of the adaptive system namely physical, interdependence, self-concept, and role function modes. In the development of NENA-q, each construct was identified under four modes of adaptation and categorized into three main constructs: organizational contribution, academic institution contribution, and personality disposition. The measured construct was described under the four adaptive modes in the following ways:

- **Interdependence mode:** it is related to the interaction of people in giving and receiving value or support in facilitating the adjustment process;
- **Role-function mode:** this mode focuses on the role of the workplace organization in mediating the role, position, and work requirements of new nurses in a society or a community;
- **Self-concept mode:** it refers to the psychological, emotional, and spiritual characteristics and personal values nurtured in a nurse;
- **Physiological physical mode:** This mode is concerned with the way new nurses interact with the organizational culture.

In addition, organizational socialization theory is also applied in this study to measure the adaptive behavioral outcome. In the third phase of the model, satisfaction, performance, turnover rate, and commitment outcomes are directly correlated to the adjustment process of new nurses [3]. Therefore, this study uses job satisfaction and competency questionnaires as proxies to measure the capability of the new nurses to adapt to the work environment. Studies have shown that new nurses who are satisfied with their current job and able to perform the nursing practices have reduced intention to leave the job [2,10,11]. In line with that, this study uses the questionnaires adapted from the Clinical Competency Questionnaires (CCQ) [12] and the Job Satisfaction Scale for Nurses [13] to measure the adaptive behavior response among the new nurses.

Finally, the NENA-q was developed in the Malay language and was based on the Roy Adaptation Model (RAM) and organizational socialization theory. It was developed to measure the factors that facilitate the adjustment process among the new nurses hired by the MOH in Malaysia (Supplementary Table S1). Exploratory Factors Analysis (EFA) was the process used to validate the NENA-q. Nevertheless, CFA was needed so that the measurement model’s relationships could be verified; CFA also ensured that the suggested theoretical model was sufficiently valid, reliable, and fit for collecting the data [14]. Given the benefits offered by CFA, the current study aimed to use this form of analysis to assess the validity of the measurement model. The purpose was to empirically prove the construct validity of various aspects that contribute to the ways newly hired nursing staff in Malaysia experience the process of adjustment.

## 2. Materials and Methods

### 2.1. The Target Population for the Study

The target population for this study was new nurses working in hospitals under MOH in 2019. Their service experience falls between 1 and 2 years. A liaison officer from the District Health Office was appointed by the nursing board to provide assistance related to the data collection. The study obtained a sampling frame of 806 eligible nurses from state hospitals nationwide. Simple probability sampling was used to choose the participants at random from the sampling frame. A collection of surveys was provided to the chosen participants, who could respond at a time convenient to them without feeling fearful or pressured.

### 2.2. Sample Size and Method of Sampling

The sample size required for validating the measurement model through CFA has become a concern for the authors, but the existing studies do not agree on the necessary CFA sample size. Usually, the minimum sample size required for small indicators is 100–150 samples [14,15,16], while some reported requiring between 250 and 500 respondents to achieve precise analysis for CFA [17,18,19]. Some authors recommended using the following guidelines regarding the sample size requirement: (a) ten cases per observation/indicator [20]; (b) one hundred cases/observations per group for multi-group modeling [21]; or (c) a sample size to parameter number ratio of five or ten [22].

Conclusively, researchers decided to use five times the number of indicators in the questionnaire since the number of indicators for latent variables is large [22,23]. As the questionnaires have 123 items, the total sample size required is 615. The researchers decided to distribute the questionnaires to the 615 randomly selected newly employed nurses. From 615, a total of 496 newly employed nurses agreed to participate, and the data were assessed for CFA.

### 2.3. Measurement Model

The four constructs, namely, OC, AIC, PT, and NENA, are reflective, with a total of 101 measurement items after EFA. These four first-order constructs formed the second-order construct.

This study intends to address the following research questions: (1) do the seven first-order constructs, namely superior role (SR), organizational support and teamwork integration (OS_TI), socio-emotional support (SES), job discriminant (JD), work readiness (WR), role function (RF), and work characteristic (WC) provide a proper measure for the second-order construct, namely organizational contribution?; (2) do the five first-order constructs, namely hardiness (HR), proactive values (PRO), self-esteem (SE), caring (CG), and optimism (OP) provide a proper measure for the second-order construct, namely personality traits?; (3) do the four first-order constructs, namely clinical component during the nursing program (CCNP), learning environment (LE), teacher characteristic (TC), and clinical teacher role (CTR) provide a proper measure for the second-order construct, namely academic institution contribution?; and (4) do the four first-order constructs namely work performance (WP), job satisfaction (JS), work commitment (CO), and self-belief (SB) provide a proper measure for the second-order construct namely newly employed nurses’ adaptation (NENA)?

### 2.4. Validating the Measurement Model of Latent Constructs

Two approaches can be employed to validate measurement models: pooled and individual CFA [24,25,26]. First of all, the CFA procedure was conducted separately for the OC, AIC, PT, and NENA constructs to assess the unidimensionality, validity, and reliability of their respective measurement models. Upon completion ofindividual CFA, all latent constructs were linked together into a single measurement model using the double-headed arrow to be validated at once through the pooled-CFA procedure. The item deletion process, respecification, and measurement of validity and reliability need to be computed prior to modeling the success factors of the NENA model.

The acceptable loading factor for each latent construct is a determination to ensure that unidimensionality is achieved [24,27,28]. If an item could not be fitted into the measurement model because of low factor loading, it was eliminated. The cut-off value for acceptable factor loading depends on the purpose of the research. However, a threshold value of 0.5 [25,29,30] is being used in this study to minimize the deletion of items.

Convergent validity was evaluated by computing the AVE for each construct. Once the AVE reaches the ≥0.5 threshold value, the constructs have attained convergent validity [14,29]. The AVE square root was then compared with the correlation coefficient between the constructs to evaluate their discriminant validity. This form of validity is attained by a construct if the squared AVE value exceeds that of the inter-construct correlation coefficient of any two constructs.

The measurement model’s fitness indices were used to assess construct validity [24,26,27]. Composite reliability (CR) [31] measures the extent to which the underlying variables of a construct are used in structural equation modeling. According to Nunnally et al., for a latent construct to achieve internal consistency and composite reliability, the CR must be ≥0.6 [23]. A high CR ≥ 0.6 value indicates that all items are constantly measuring their respective constructs.

### 2.5. The Fitness of a Measurement Model

Among scholars, different fitness indices have been reported in the articles. The recommendation of Brown was to report fit indices as absolute fit (chi-squared goodness-of-fit [χ^2^] and standardized root mean square residual, or SRMR), parsimony-corrected fit (root mean square error of approximation, or RMSEA), and comparative fit (Tucker–Lewis fit index [TLI] and comparative fit index [CFI]) [32]. They recommended using at least one index from the three fitness categories, which are absolute fit, incremental fit, and parsimonious fit [26,27,33]. However, there are no restrictions on scholars to select the types of fitness index that should be reported, as it refers to the method of articles referred to by the author. The current study utilized common fitness index used by many scholars: chi-squared over degree of freedom (Chisq/df), Root Mean Square Error Approximation (RMSEA), and Comparative Fit Index (CFI) [34]. A model fit was indicated using a set of cut-off values: the RMSEA values were to be from 0.05 to 1.00; the CFI ≥ 0.90; and the Chisq/df ≤ 5.00. This would indicate a reasonable fit, as stated by Moss et al. [35].

## 3. Results

Two types of measurement models, namely, the original model and the new model, are presented in Table 1. The outcomes from the exploratory factor analysis (EFA) were used as the basis for developing the original model. The new model is the final model after the deletion process to achieve the unidimensionality requirement and fulfill the required values of fitness indices. Therefore, the new models have fewer indicators compared to the original model. The convergent validity, composite reliability, and construct validity are summarized in Table 2.

### 3.1. Construct Validity, Convergent Validity, and Composite Reliability

#### 3.1.1. Assessment of the Organization Contribution (OC) Model

The measurement model for the OC construct had achieved construct validity as the fitness indices achieved the thresholds of the model fit categories. Initially, checking is done on the model by deleting the poor items. Three items (C1_WR, C3_JD, and C4_JD) were removed from the construct due to low factor loading. The lowest factors were deleted first, and the deletion process was performed one item at a time to prevent missing any optimal result [25]. Having deleted certain items, all the remaining items’ factor loadings were seen to be adequate, as they ranged between 0.5 and 0.7. The indication was that each variable belonged to the latent construct that would then be assessed [36]. Additionally, the items that could best explain the constructs were those with the highest factor loadings. Next, redundant items (C1_OSTI and C1_SR) were identified through modification indices (MI) produced by the algorithm.

Convergent validity was confirmed when the AVE surpassed the threshold of 0.5. However, from the seven subconstructs of the new OC model, only one construct (job discrimination) has an AVE value less than 0.50. The researchers decided to eliminate the construct as it was assumed that the variables did not contribute to measuring job discrimination in the perceptions of newly employed nurses. Reflective factors comprised the second-order construct, so deleting the sub-construct was not a problem as sufficient factors remained to assess the organization’s role in enabling the newly qualified nursing staff to adapt more easily to the transition stage [37]. After deletion, the fitness indices indicate CFI = 0.91, CMIN/df = 4.06, and RMSEA = 0.08. The organizational construct that has six sub-constructs managed to achieve the limit of 0.6 for CR.

#### 3.1.2. Assessment of the Academic Institutions Contribution (AIC) Model

Academic institutions’ contributions consist of four latent constructs: clinical component of nursing program (CCNP), learning environment (LE), teacher characteristics (TC), and clinical teacher role (CTR), and comprise a total of 15 items to be measured. Similar steps were conducted for the academic institutions’ contribution model to achieve the best fit index for the measured construct. The findings indicated that the correlations between the variables and the factors used were higher than 0.5. This suggested the practical significance and feasibility of each item and factor. Meanwhile, satisfactory levels of convergent validity were achieved as the four constructs’ AVE values were higher than 0.5. The results for the model fitness indexes were satisfied in the original measurement model of academic institution contribution (Chisq/df = 3.74, RMSEA = 0.07, and CFI = 0.96) and thus do not require any modification to the original model.

#### 3.1.3. Assessment of the Personality Traits (PT) Model

The measurement model consists of 24 items under five first-order constructs or components, namely hardiness, proactiveness, self-esteem, caring, and optimism, which were coded as personal traits. Initially, the factor loading for each item was above 0.5. However, convergent validity was not achieved when the AVE was less than 0.5, according to the validity test. Therefore, D4_HR items that had the lowest factor loading were removed. After dropping one item measuring hardiness, the AVE value for all five latent constructs achieved the threshold value. However, the model is still considered a poor fit because it does not meet the threshold value. Next, redundant items (D3_OP) were identified through modification indices (MI) produced by the algorithm. At this point, fit indices improve with CFI = 0.92, Chisq/df = 4.01, and RMSEA = 0.08.

#### 3.1.4. Assessment of the Newly Employed Nurses Adaptation (NENA) Model

The EFA procedure extracted four components. The first component was renamed as work performance (WP). This component consists of 11 items, which explained 30.8% of the variance with factor loadings ranging between 0.70 and 0.91. The second component was renamed as job satisfaction (JS). This component consisted of 8 items, which explained 16.9% of the variance with factor loadings ranging between 0.69 and 0.89. The third component was renamed as work commitment (CO). This component consisted of seven items, which explained 16.4% of the variance with factor loadings ranging between 0.54 and 0.84. The fourth component was renamed self-belief (SB). This component consisted of three items that explained 14.1% of the variance, with factor loadings ranging between 0.67 and 0.81. The total variance explained for the construct was 78.2%, indicating that the instruments were successful in measuring the NENA model.

In CFA analysis, the measurement model consisted of four first-order factors: work performance, job satisfaction, work commitment, and self-belief, and was coded as the NENA model. The assessment was carried out on the NENA construct by deleting the redundant items suggested by the modification index (MI). The redundant items identified through MI (WP22) were removed. The measurement model has achieved the required fitness indices, namely RMSEA = 0.07, Chi-Square/Df = 3.62, and CFI = 0.91.

#### 3.1.5. Assessment of the Pooled CFA and Discriminant Validity for the NENA-q Model

**Figure 1:**
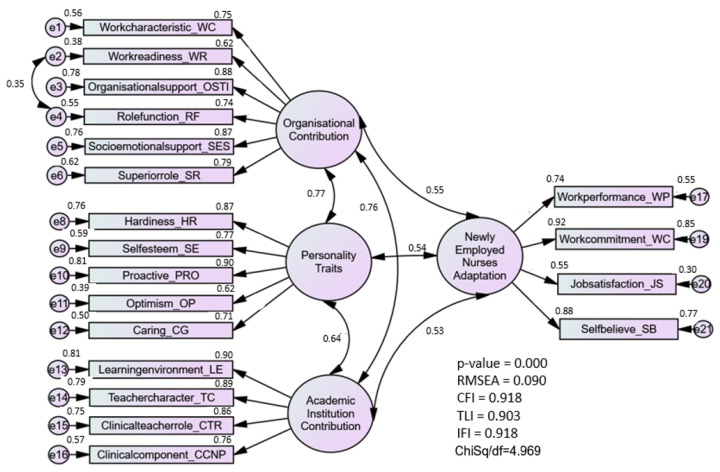
The four constructs are assessed together through a pooled-CFA procedure.

In the pooled CFA, the complexity of the model was reduced and simplified. The pooled-CFA has merged four constructs, as shown in Figure 1, which consist of OC, PT, AIC, and NENA. The main reason to run pooled CFA is to ensure discriminant validity among the constructs in the model. The result shows that the factor loading for each sub-construct is greater than 0.5 and the correlation coefficient between two latent constructs does not exceed 0.85. This indicated that there were no multicollinearity issues in the model. The pooled-CFA model for NENA-q is presented in Figure 1, which indicates that the model fitness indexes were satisfied in the final measurement model of public leadership (Chisq/df = 4.97, RMSEA = 0.09, and CFI = 0.92).

Subsequently, the construct pairs’ correlation coefficients were assessed to determine the discriminant validity of each construct. As presented in Table 3, the matrix’s diagonal values illustrate the four latent constructs’ square roots of AVE; the remaining values show the correlations between each construct pair. If the diagonal value was higher than those in the row or column, then discriminant validity had been attained. The study found all four constructs in the model fulfilled the criteria of discriminant validity.

## 4. Discussion

### 4.1. Academic Institution Contribution (AIC) Construct

The proposed AIC model fits well without any further deletion processes in the CFA analysis. Extensive removal of items with low factor loading has been done in the EFA stage. All items in the AIC model, which comprise the learning environment, teacher characteristics, a classroom component in the nursing program, and the clinical teacher role were retained.

The classroom component in the nursing programs sub-construct explores the perception of newly graduated nurses on their experience during the academic preparation to prepare them for practice in a real-life setting. Research from Canada indicated that preparing a new graduate to safely practice nursing care requires a generalist foundation as well as particular skills and capabilities linked to the job [38]. Even though the new graduates believe that they are ready and have enough knowledge, it is different from the nursing preceptor’s perspective. They reported that the new nurses are not sufficiently prepared and are not confident in delivering their skills and capabilities for practice [39]. An Australian study has described several measures to prepare and improve the preparation for practices in real-life settings, such as having an effective mentor during practicum, and educators should provide guidance and support to improve knowledge and master what is required in clinical skills [40]. In addition, a close connection between clinical mentors and academic staff should improve the development of students’ familiarity with the hospital’s environment, and improve their feeling of being supported and this will slowly develop the self-confidence among nursing students’ readiness to practice [9].

The active engagement of student nurses in their learning activities can be sustained and promoted using various teaching strategies and styles [9]. The use of stimulating teaching approaches improves academic performance, helps learners contextualize theoretical class-based aspects when encountered in practice, facilitates proactive searches of various research works, and likely leads to more questions [41,42]. The AIC model is consistent with the Faculty Attributes with Confidence, Equilibrium, and Success (FACES) Theory, which was developed to contextualize the nursing educators’ responsibilities and behaviors to ensure the new graduates are well prepared themselves and adapt quickly in the transition phase [43]. Therefore, the authors perceived teacher characteristics, the clinical teacher role, and the learning environment as important measures in determining the contribution of academic institutions in facilitating the adaptation of newly graduated nurses.

### 4.2. Organizational Contribution (OC) Construct

The initial measurement model of OC has emerged as a first-order construct consisting of seven components. These are the characteristics of work (WC), the role of superiors (SR), support from the organization, and the integration of teamwork (OS_TI), socio-emotional assistance (SES), being ready to work (WR), role function (RF), and discrimination in the job (JD). All the components were assessed using a specific number of questionnaire items. Based on CFA, if an item had a factor loading below 0.5 or a high value on a modification index, it was removed from the construct.

Nevertheless, having deleted certain items, it was revealed through the model that although the fitness indices of the first measurement model using the OC construct had achieved the necessary model-fit level, the job discrimination component AVE was identified as being under 0.5. Initially, researchers perceived the component of job discrimination as being used to measure the ‘acceptance of organizational insiders’ based on the organizational socialization theory [44]. However, due to the offending factor loading, the job discrimination sub-construct was eliminated from the OC measurement model, leaving only six sub-constructs. Thus, the validity and reliability of the model were enhanced by removing the component of job discrimination, as this made no contribution to assessing the construct and would not be relevant when practicing the job [45]. The respecified measurement model indicates that the factor loading for items in the organizational construct is greater than 0.5, and fitness indices were satisfied in the final measurement model of organizational contribution.

Interventions involving mentorship and fostering help a newly registered nurse in terms of how they develop and socialize in organizations or units [46]. Mentoring or orientation programs previously proved that the programs could provide opportunities for new graduates to adapt to new environments and gain a better understanding of work culture [47,48,49]. In addition, with the constant guidance of an experienced nursing professional by their side, new nurses can gain the confidence to overcome challenges during this transition period and at the same time will be able to develop new skills [8,50,51].

An assumption is normally made about the clarity of the role, according to the information obtained regarding the expectations, accountability, and scope of professional positions [52]. If they are to promote better-developed role clarity, all workplace-based entities need to outline the roles and give all the essential instructions [53], as well as promote collaboration among staff [54]. This would enable a new nurse to gain a definite knowledge of the tasks, obligations, and work procedures [55]. Role clarity is a predictor of job satisfaction, work performance, and a low turnover rate [44,56,57]. It is important to avoid any misunderstanding between new nurses and other workers’ responsibilities, and thus provide delegation and prioritization skills for new nurses [54]. Furthermore, according to the framework of organizational socialization theory, role clarity is regarded as an important mediator of the adaptation phase as well as an indicator of how well individuals socialize within an organization [44]. Therefore, the role of the organization in assisting new graduates to understand the required standards, job scope, responsibilities, guidelines, and work protocols is important in supporting the socialization process.

An Australian study shows that work readiness involves a professional role and has an impact on new graduates’ job satisfaction, work commitment, and retention rate [58]. There are four dimensions of work readiness, which involve personal, clinical, relational characteristics, and organizational acuity [59]. Nevertheless, organizational acuity is referred to in the work readiness component of the NENA-q. This refers to newly graduated nurses’ ability to self-direct and motivate themselves in order to achieve predictability at work, be prepared to work, and successfully complete tasks without difficulty or obstacle while achieving the best possible outcomes from specific targets [60].

### 4.3. Personality Traits (PT) Construct

The proposed PT construct fit well after the removal of only one item from the hardiness component. A five-factor component was characterized by five attributes, namely hardiness, optimism, self-esteem, caring value, and proactive attitude, consisting of 22 items obtained at the end of the individual CFA.

The concept of hardiness reflects a large extent of personal potential to cope with and overcome a stressful and difficult life situation with the aim of achieving socio-psychological adjustment [61]. Hardiness traits are considered to be a stress-relieving and happiness-promoting factor in nurses [62]. Researchers feel the value of hardiness should be assessed in new nurses since hardiness can be viewed as a personal resource that allows individuals to utilize contextual factors such as work motivation to reduce depression or improve psychological well-being [63].

In addition, it has been proven empirically that a newly graduated nurse with a proactive character develops in a hardier way, has increased confidence in themselves, and copes better in any stressful situation; turnover is also reduced [9,53]. Consistent with this, the engagement of proactive behaviors among newly graduated nurses contributes to the development of hardiness to overcome challenges and resistance [53,64]. In addition, caring is an essential component of human relationships and is a key foundation in the nursing profession [65]. The core values in the nursing profession include human dignity, integrity, autonomy, altruism, and social justice, which are required for nurses to integrate with caring [66]. Caring includes sensitivity, empathy, loving, general concern, genuine interest, kindness or compassion towards patients and members of the healthcare team [67]. Studies reported that nurses who cultivate a caring attitude indirectly have strong self-resilience as they are able to develop an inner potential to handle the external environment well and politely [68].

Added to that, optimism and self-esteem were conceptualized as personal indicators of adaptivity because they mediate the relationship between environmental factors and the individual’s self-concept [69]. In nursing undergraduates’ studies, career motivation has a relatively positive association with optimism [69]. To gain competencies, newly qualified nursing staff need to be optimistic, have self-esteem [9], be able to surmount problems, and show resilience to stimuli from inside and outside [70]. Thus, it can be said that self-efficacy and optimism have a correlation with behavioral and emotional control through the mediation of basic cognitive structures and processes.

### 4.4. Newly Employed Nurses Adaptation (NENA) Construct

Organizational socialization theory was applied in this study to measure the adaptive behavioral outcome [3]. In the third phase of the model, four outcomes, namely job satisfaction, work performance, turnover rate, and commitment, are directly correlated to the adjustment process of new nurses [44]. Initially, this study used job satisfaction and competency questionnaires as proxy measures to assess the level of adaptation among new nurses. Taking into account, a previous study reported that new nurses who are satisfied with their current job and able to perform the nursing practices are highly correlated with the intention to stay inthe profession [28]. In this study, the intention to leave was measured as the moderator for the SEM analysis. Therefore, this study uses the questionnaires adapted from the Clinical Competency Questionnaires (CCQ) [12] and the Job Satisfaction Scale for Nurses [13] to measure the adaptive behavior responses among new nurses. Through EFA analysis, on each of the 33 items, principal component analysis featuring varimax (25) rotation was performed. Four was established as the component number to code into the NENA model: performance at work, satisfaction with the job, how committed they were, and how much they believed in themselves. In the CFA analysis, after four items were removed, all four latent constructs had a factor loading of more than 0.5. At this point, the value of AVE is greater than 0.50 and the value of CR exceeds 0.6.

### 4.5. Pooled-CFA of Newly Employed Nurses Adaptation (NENA-q) Model

A pooled CFA for NENA-q model could be run simultaneously after the CFA for each individual models is computed with the assessment forunidimentionality, validity and reliability. This model is considered complicated when it involves many second-order constructs and items. Therefore, pooled CFA is recommended to be performed on a complex model to make it easier to analyze and understand. In addition, the purpose of pooled-CFA was to assess the discriminant validity between constructs in the models [25,26,33], from the pooled-CFA analysis, it can be concluded that the three main components influencing the adaptation of new nurses in Malaysia are workplace organization, the role of an academic institution, and individual personality.

A good fit was noted for the NENA-q model, while each fitness index category had been satisfied following modification, which led to the removal of 10 (or 9.9%) of the 101 items. It was surprising that the discriminant outcomes showed that every parameter was different from the other constructs. When the correlation value is lower than the square root of AVE, discriminant validity is attained for a model. It indicates that each component of the construct was measured independently and is unrelated to one another. From the results, we can say that the new graduate nurse’s adjustment process during the transition phase is dependent on the nurses’ coping resources and organizational roles, as well as the active involvement of academic institutions in preparing new graduates to practice as professional nurses.

## 5. Conclusions

In this context, newly employed nurses were an open system comprised of adaptive modes, namely interdependence, self-concept, role function, and physiology adaptive mode. The components of Teacher Characteristic (TC), Clinical Teacher Role (CTR), and Superior Role (SR) were categorized as interdependence adaptive modes. The items in the component are related to the interaction of teachers, superiors, or other nursing staff in providing values or support in assisting new nurses in their adjustment process. The component that focuses on the role of nursing academic and workplace organizations in mediating the role, position, and work requirements of new nurses in a society or community were categorized as a role-function adaptive mode. It includes the components of Learning Environment (LE), Clinical Component of Nursing Programs (CCNP), Work Characteristic (WC), Organizational support and teamwork integration (OS_TI), Work Readiness (WR), and Role Function (RF). The items measured in the Socio-Emotional Support (SES) component were concerned with the way new nurses interact with the organizational culture, and thus, it was categorized as the physiology adaptive mode. All components in the PT, namely hardiness (HR), self-esteem (SE), optimism (OP), caring (CG), and proactive (PRO) values, were categorized as self-concept adaptive modes. In order to measure the adaptive outcome, four constructs, namely Work Performance (WP), Job Satisfaction (JS), Commitment (CO), and Self-Believe (SB), were used as proxies to measure the adjustment level of newly employed nurses in Malaysia.

Overall, the NENA-q model consisting of OC construct, AIC construct, PT construct, and NENA construct for CFA has met the fitness indexes and passed the measurements of the AVE, CR, and normality test. These CFA results will be used to model structural equation modeling (SEM) for further path analysis. The knowledge derived from this study may encourage collaboration between nursing academic institutes and workplace organizations to guarantee that new nurses always have access to updated knowledge in their specific clinical field. In addition, the measurement model indicated a requirement of both institutions to develop, emphasize, and strengthen a nurse’s personality through continuous programs or training to assure that a new nurse possesses the personality qualities required to fit into their new working environment.

### Limitation

Malaysia is experiencing a nursing shortage. Starting in 2011, MOH has started absorbing new nurses from private academic institutions. The number of privately graduated nurses hired in the MOH facilities is increasing from year to year. Nonetheless, the study had the limitation of only including nurses who had recently graduated from a public academic institution (a Ministry of Health training center). There are different approaches, especially in teaching mechanisms and academic culture, between private and public nursing academic institutions. Therefore, future studies should consider new nurses who graduated from both public and private academic institutions, using the same research questions and mixed-method research design to compare the variation in the contributing adaptation factors and identify the new emerging factors between both institutions.

In addition, only new nurses who are working in general hospitals are involved in this study, as the number of hired nurses in 2019 is high in general hospitals compared to other types of hospitals. In the future, it is recommended to expand this study to assess new nurses working in other MOH facilities, such as district hospitals, specialized hospitals, military hospitals, and health clinics, to make it more generalizable.

## Figures and Tables

**Table 1:** Individual CFA of the original and new measurement models.

Original Model	New Model
**Organisational Contribution (OC)**	
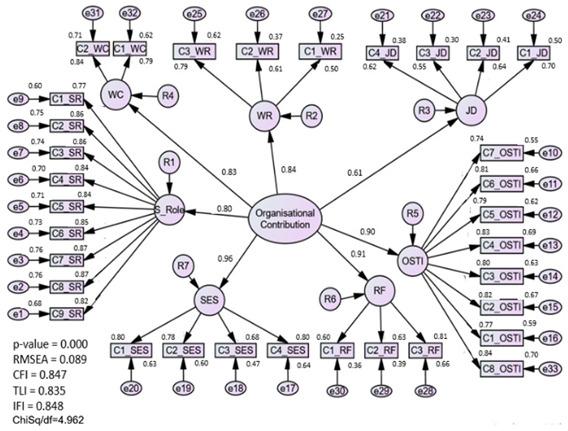	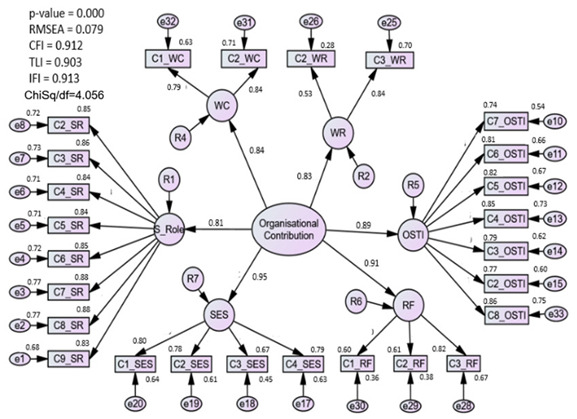
**Academic Institution Contribution (AIC)**	
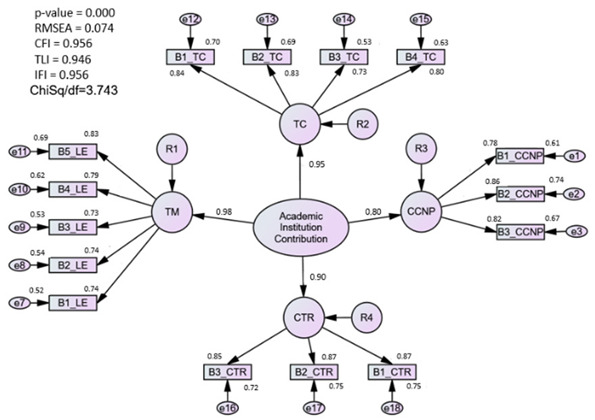	No modification required as the convergent and Composite reliability achieved for the original model.
**Personality traits (PT)**	
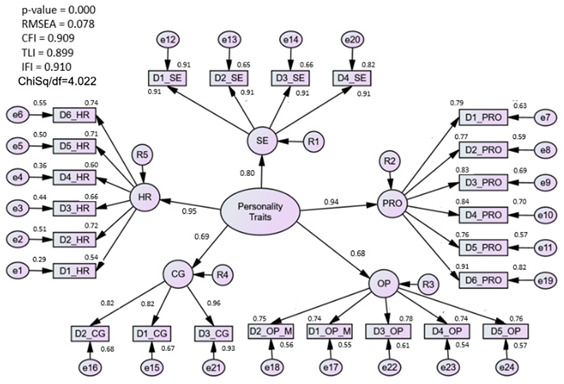	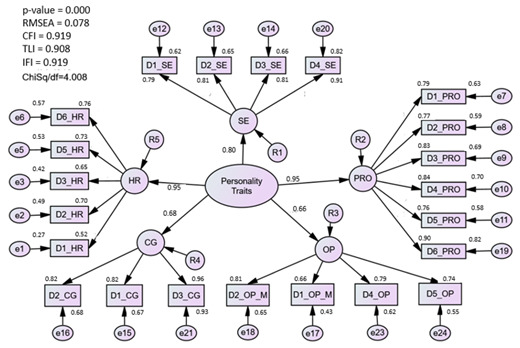
**Newly Employed Nurses Adaptation (NENA)**	
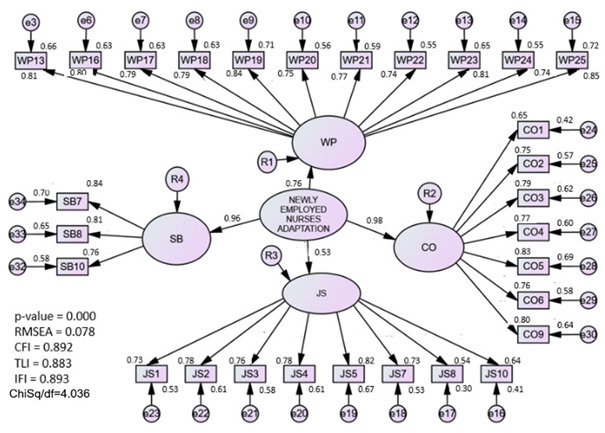	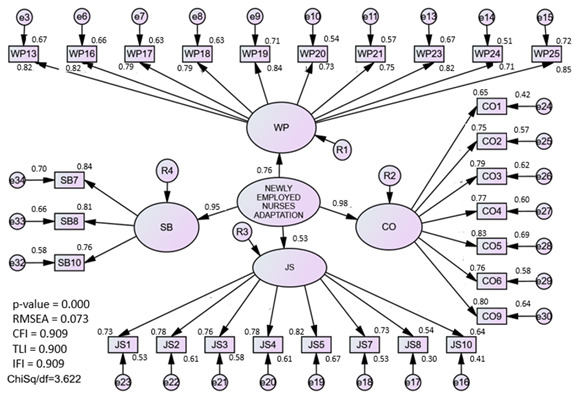

**Table 2:** The CFA report for construct validity, convergent validity, and composite reliability.

Components	Convergent Validity	Composite Reliability	Construct Validity		
	AVE	CR	RMSEA	Chisq/df	CFI
**Organisational Contribution**					
Superior Role	0.727	0.975	0.079	4.056	0.912
Organisational support & teamwork integration	0.652	0.959			
Socio-emotional support	0.584	0.907			
Work Readiness	0.491	0.747			
Role function	0.470	0.811			
Work Characteristic	0.666	0.879			
**Academic Institution Contribution**					
Class/clinical components during nursing programs	0.677	0.920	0.074	3.743	0.956
Learning Environment	0.582	0.924			
Teacher characteristics	0.639	0.927			
Clinical teacher role	0.738	0.940			
**Personality traits**					
Hardiness	0.457	0.872	0.078	4.008	0.919
Proactive	0.667	0.956			
Caring	0.758	0.945			
Optimism	0.562	0.899			
Self-Esteem	0.689	0.941			
**Newly Employed Nurses Adaptation**					
Work Performance	0.632	0.968	0.073	3.622	0.909
Job satisfaction	0.530	0.938			
Work Commitment	0.587	0.946			
Self-Belief	0.647	0.908			

**Table 3:** The discriminant validity index summary.

Construct	Newly Employed Nurses Adaptation	Organisational Contribution	Personality Traits	Academic Institution Contribution
**Newly Employed Nurses Adaptation**	**0.786**			
**Organisational Contribution**	0.546	**0.780**		
**Personality traits**	0.540	0.773	**0.782**	
**Academic Institution Contribution**	0.526	0.760	0.637	**0.854**

## Data Availability

The data that support the findings of this study are available on request from the corresponding author, [Aniza Ismail]. The raw data are not publicly available due to restrictions [because they contain information that could compromise the privacy of research participants].

## Supplementary Materials

The following supporting information can be downloaded at: https://www.mdpi.com/article/10.3390/ijerph20042860/s1, Table S1: Questionnaire on determinants influencing effective transition adaptation behavior of newly employed nurses in ministry of health hospitals nurses in ministry of health hospitals.
