# Supplementary material for: Validating an Instrument for Measuring Newly Graduated Nurses’ Adaptation
Source: Int J Environ Res Public Health. 2023 Feb 6;20(4):2860. doi: 10.3390/ijerph20042860 (PMC9957435; doi:10.3390/ijerph20042860)
Supplement: Supplementary file 1 [file ijerph-20-02860-s001.zip › ijerph-2189122-supplementary.pdf]


For office use only

QUESTIONNAIRE ON DETERMINANTS INFLUENCING EFFECTIVE TRANSITION ADAPTATION BEHAVIOUR OF NEWLY EMPLOYED NURSES IN MINISTRY OF HEALTH HOSPITALS NURSES IN MINISTRY OF HEALTH HOSPITALS

SECTION A: GENERAL INFORMATION

A001 Age: ______________________

A002 Gender

1. Man ☐

2. Women ☐

A003 Ethnicity: ______________________

A004 Religion: ______________________

A005 Marital Status:

Never married ☐

Married ☐

Divorced ☐

Cohabiting ☐

A006 No. of children: ____________

SECTION A1: EMPLOYMENT INFORMATION, BOTH PREVIOUS AND CURRENT

A007	Grade and profession	________________				
A008	Place of Work	________________				
A009	Department or Unit	________________				
A010	When did you start serving in the MOH?	________Month_______Year				
A011	When did you start serving in the current department or unit?	________Month_______Year				
A012	When have you graduated?	________Month_______Year				
A013	Period of unemployment after graduation	________Month_______Year				
A014	Do you have work experience after graduation?	Yes	☐	No	☐	
	If Yes, state your field of work	Health Sector	☐	Non-health Sector	☐	
A015	Do you intend to resign from your job?	Yes	☐	No	☐	

Section B: NURSING EDUCATION PROGRAM				
**1**	**2**	**3**	**4**	**5**
Strongly disagree	*Disagree*	*Neither Disagree nor Agree*	*Agree*	*Strongly agree*

**B1: Learning Environment**						
B001	Staff nurses facilitate me in during the practical.	1	2	3	4	5
B002	I am accepted as a learner, encouraged to ask questions in contributing to patient/client care	1	2	3	4	5
B003	Members of the multidisciplinary team contribute to my learning experience	1	2	3	4	5
B004	Instructors used teaching aids to assist student's understanding in the class	1	2	3	4	5
B005	Instructors able to explain well on the teaching topic	1	2	3	4	5
**B2: Classroom Component During Nursing Program**						
B006	The classroom component of my nursing education programme adequately prepared me to recognise the desired actions, side effects, and interactions of medications.	1	2	3	4	5
B007	The classroom component of my nursing education programme adequately prepared me to analyse multiple types of data when making client-related decisions.	1	2	3	4	5
B008	The classroom component of my nursing education programme adequately prepared me to understand the pathophysiology underlying clients' conditions.	1	2	3	4	5
**B3: Teacher Characteristic**						
B009	The instructors help me link theory with clinical practice.	1	2	3	4	5
B010	The instructors keep professionals with students	1	2	3	4	5
B011	The instructors were rigid in rule implementation.	1	2	3	4	5
B012	The instructor discusses my learning needs with me.	1	2	3	4	5
**B4: Clinical Teacher Role**						
B013	The Clinical Facilitator assisted in my interactions with unit staff to ensure that I was provided with timely and constructive feedback.	1	2	3	4	5
B014	The clinical facilitator, through negotiation with the unit staff, helped me achieve my learning goals.	1	2	3	4	5
B015	The clinical facilitator showed genuine concern for my progress and needs.	1	2	3	4	5

Section C: WORKPLACE ORGANISATION				
**1**	**2**	**3**	**4**	**5**
Strongly disagree	*Disagree*	*Neither Disagree nor Agree*	*Agree*	*Strongly agree*

**C1: Superior/Mentor Role**						
B001	My supervisor treats me and my colleagues fairly.	1	2	3	4	5
B002	My supervisor distributes the work fairly and impartially	1	2	3	4	5
B003	If needed, I can get support and help from my supervisors	1	2	3	4	5
B004	My supervisor encourages me to develop my nursing skills	1	2	3	4	5
B005	My supervisor tackles problems as soon as they surface.	1	2	3	4	5
B006	My supervisor is concerned about the welfare of the subordinates.	1	2	3	4	5
B007	My supervisors and doctors support me when I take appropriate risks, even those that fail.	1	2	3	4	5
B008	My supervisor gives me help and support in getting the job done.	1	2	3	4	5
B009	My supervisor treats my mistakes as opportunities for me to learn and improve.	1	2	3	4	5
**C2: Organisational Support and Teamwork Integration**						
B010	This organisation is a great place to work	1	2	3	4	5
B011	This organisation really inspires me to give my very best job performance	1	2	3	4	5
B012	The training and development I've received have improved my performance.	1	2	3	4	5
B013	I am participating in the working group or in a team.	1	2	3	4	5
B014	Working in the group helps you learn more about nursing practice.	1	2	3	4	5
B015	The work environment always supports my duty and its execution.	1	2	3	4	5
B016	I am satisfied with the work I do	1	2	3	4	5
B017	Workers are encouraged to think of ways to do things better at the workplace.	1	2	3	4	5

Section C: WORKPLACE ORGANISATION				
**1**	**2**	**3**	**4**	**5**
Strongly disagree	*Disagree*	*Neither Disagree nor Agree*	*Agree*	*Strongly agree*

**C3: Socioemotional Support**						
B018	There is sufficient communication in my department	1	2	3	4	5
B019	If needed, I can talk with my friends about work-related problems	1	2	3	4	5
B020	My supervisor gives me enough feedback to help me improve my clinical practice	1	2	3	4	5
B021	I am satisfied with my ability to solve problems at work	1	2	3	4	5
**C4: Job Discrimination**						
B022	The discrimination in my office affects my job and job skills.	1	2	3	4	5
B023	Harassment and discrimination are prevented at my workplace	1	2	3	4	5
B024	The work load affects my job and skills.	1	2	3	4	5
B025	My colleague's interference affects my job and skills.	1	2	3	4	5
**C5: Work Readiness**						
B026	I know in advance what kind of tasks to expect a month from now	1	2	3	4	5
B027	I feel it is necessary to demonstrate my ability and competence to others in order to be assigned to attractive tasks or projects	1	2	3	4	5
B028	I know what has to be learned and which new skills have to be acquired in order to gain skills in my job	1	2	3	4	5
**C6: Role Function**						
B029	My job is routine every day	1	2	3	4	5
B030	I have the ability to choose what and how I do my work.	1	2	3	4	5
B031	I am clear about the scope of work, goals, and objectives in my work	1	2	3	4	5
**C7: Work Characteristic**						
B032	Any inequalities at my workplace have been addressed.	1	2	3	4	5
B033	Usually my working hours extend beyond the actual working hours.	1	2	3	4	5

Section D: PERSONALITY TRAITS				
**1**	**2**	**3**	**4**	**5**
Strongly disagree	*Disagree*	*Neither Disagree nor Agree*	*Agree*	*Strongly agree*

**D1: Proactive**						
B001	I try to pinpoint what I need to succeed.	1	2	3	4	5
B002	After attaining a goal, I look for another, more challenging one.	1	2	3	4	5
B003	I always try to find a way to work around obstacles; nothing really stops me.	1	2	3	4	5
B004	When I encounter a problem, I take the initiative to solve it.	1	2	3	4	5
B005	Changes in routine are interesting to me	1	2	3	4	5
B006	If someone tells me I can't do something, I can be sure I will do it.	1	2	3	4	5
**D2: Hardiness Scale**						
B007	Things go in my life depends on my own actions	1	2	3	4	5
B008	The majority of my time is spent doing meaningful things.	1	2	3	4	5
B009	By working hard, I can nearly always achieve my goals	1	2	3	4	5
B010	In uncertain times, I usually expect the best.	1	2	3	4	5
B011	Most days, life is really interesting and exciting for me	1	2	3	4	5
B012	I'm always optimistic about my future.	1	2	3	4	5

Section D: PERSONALITY TRAITS				
**1**	**2**	**3**	**4**	**5**
Strongly disagree	*Disagree*	*Neither Disagree nor Agree*	*Agree*	*Strongly agree*

**D3: Optimism**						
B013	I don't like to make changes in my regular activities	1	2	3	4	5
B014	I hardly ever expect things to go my way.	1	2	3	4	5
B015	At times, I think I am no good at all	1	2	3	4	5
B016	I often find it difficult to express empathy with patients	1	2	3	4	5
B017	Overall, I expect more good things to happen to me than bad.	1	2	3	4	5
**D4: Caring**						
B018	I am confident in my ability to express concern for my patients.	1	2	3	4	5
B019	It is easy for me to consider the many facets of a patient's care while I am listening to them.	1	2	3	4	5
B020	I have the ability to restore a sense of normalcy in stressful situations.	1	2	3	4	5
**D5: Self-Esteem**						
B021	I feel that I'm a person of worth, at least on an equal plane with others	1	2	3	4	5
B022	I am able to do things as well as most other people	1	2	3	4	5
B023	On the whole, I am satisfied with myself.	1	2	3	4	5
B024	I wish I could have more respect for myself.	1	2	3	4	5

Section D: NEWLY EMPLOYED NURSES' ADAPTATION				
**1**	**2**	**3**	**4**	**5**
Strongly disagree	*Disagree*	*Neither Disagree nor Agree*	*Agree*	*Strongly agree*

**E1: Work Performance**						
B001	Administering intravenous medications (or into intravenous bags)	1	2	3	4	5
B002	Administering oral medications	1	2	3	4	5
B003	Administering blood transfusion	1	2	3	4	5
B004	Performing urinary catheter insertion and care	1	2	3	4	5
B005	Performing sterile techniques	1	2	3	4	5
B006	Performing postural drainage and percussion, and oxygen therapy	1	2	3	4	5
B007	Performing preoperation/postoperation care	1	2	3	4	5
B008	Performing tracheotomy care	1	2	3	4	5
B009	Performing nasogastric tube feeding and care	1	2	3	4	5
B010	Performing chest tube care with underwater seal management					
B011	Performing wound dressing care					
**E2: Job Satisfaction**						
B012	I am proud of my current job.	1	2	3	4	5
B013	The provision of nursing care is what I like to do	1	2	3	4	5
B014	I clearly understand my problems and challenges at work.	1	2	3	4	5
B015	Working in this hospital helps me improve nursing practice.	1	2	3	4	5
B016	Helping patients recover motivates me to work as a nurse.	1	2	3	4	5
B017	I can consult with other professionals on patient care.	1	2	3	4	5
B018	I sometimes receive praise and recognition from colleagues	1	2	3	4	5
B019	I can balance work with my private life.	1	2	3	4	5

Section D: NEWLY EMPLOYED NURSES' ADAPTATION				
**1**	**2**	**3**	**4**	**5**
Strongly disagree	*Disagree*	*Neither Disagree nor Agree*	*Agree*	*Strongly agree*

**E3: Work Commitment**						
B001	Following health and safety precautions	1	2	3	4	5
B002	Taking appropriate measures to prevent or minimise the risk of injury to oneself	1	2	3	4	5
B003	Taking appropriate measures to prevent or minimise the risk of injury to patients	1	2	3	4	5
B004	Adhering to the regulation of patients' and families' confidentiality	1	2	3	4	5
B005	Adhering to ethical and legal standards of practice	1	2	3	4	5
B006	Maintaining appropriate appearance, attire, and conduct	1	2	3	4	5
B007	Assisting activities and mobility, and changing position	1	2	3	4	5
**E2: Self-Bieleve**						
B011	Communicating verbally with precise and appropriate terminology in a timely manner with patients and families	1	2	3	4	5
B012	Educating patients or families with disease-related care knowledge	1	2	3	4	5
B013	Providing emotional and psychosocial support	1	2	3	4	5

Thank you for your cooperation
